# “Vis-à-Vis Training” to Improve Emotional and Executive Competences in Very Preterm Children: A Pilot Study and Randomised Controlled Trial

**DOI:** 10.3390/children11080956

**Published:** 2024-08-08

**Authors:** Maria Chiara Liverani, Vanessa Siffredi, Greta Mikneviciute, Emma Mazza, Russia Ha-Vinh Leuchter, Petra Susan Hüppi, Cristina Borradori Tolsa, Edouard Gentaz

**Affiliations:** 1 Division of Development and Growth, Department of Paediatrics, Gynaecology and Obstetrics, Geneva University Hospitals, 1205 Geneva, Switzerland; vanessa.siffredi@chuv.ch (V.S.); greta.mikneviciute@unige.ch (G.M.); emma.mazza@tele-service.be (E.M.); russia.ha-vinhleuchter@hug.ch (R.H.-V.L.); petra.huppi@hug.ch (P.S.H.); cristina.borradoritolsa@hug.ch (C.B.T.); 2 SensoriMotor, Affective and Social Development Laboratory, Faculty of Psychology and Educational Sciences, University of Geneva, 1000 Geneva, Switzerland; edouard.gentaz@unige.ch; 3 Department of Radiology, Lausanne University Hospital (CHUV), 1015 Lausanne, Switzerland; 4 Faculty of Biology and Medicine, University of Lausanne (UNIL), 1015 Lausanne, Switzerland

**Keywords:** preterm, children, socio-emotional competences, executive functions, intervention, randomised controlled trial

## Abstract

Background/Objectives: Premature birth can lead to socio-emotional, behavioural and executive problems that impact quality of life and school performance in the long term. The aim of this pilot study was to evaluate the feasibility and efficacy of a 12-week computerised training called Vis-à-vis to enhance these competencies in a cohort of very preterm (VPT) children aged 6 to 9. Methods: This pilot randomised controlled trial included 45 children born before 32 gestational weeks. Socio-emotional, behavioural and executive competencies were evaluated at three time points using computerised tasks, neuropsychological tests and questionnaires. Results: Among the eligible VPT children, 20% (n = 45) accepted to be part of the study, and 40% (n = 18) dropped out. Finally, 60% (n = 27) of the enrolled participants completed the study. Results showed a significant improvement in emotion knowledge and recognition immediately after the completion of the training. Conclusions: Overall, our results indicate that the implementation of this type of computerised training is feasible, but the overall compliance is unsatisfactory given the high dropout rate. Nevertheless, the positive effect of the training on emotion recognition encourages further exploration of these kinds of interventions to prevent adverse consequences in children born too soon.

## 1. Introduction

Prematurity is defined as a birth that occurs before 37 weeks of gestation. It represents 10.6% of births worldwide, and this number is increasing constantly [1]. Children born preterm are particularly vulnerable to physical, psychological and emotional difficulties, and these fragilities last until adulthood [2,3]. They have a two to four-fold higher risk of having psychopathological symptoms in comparison to term-born peers [4], and the severity of the diagnosis increases with each decreasing gestational week at birth [5]. In their review, Johnson and Marlow [6] defined the “preterm behavioural phenotype”, which summarises the typical pattern of emotional, cognitive and behavioural difficulties specific to this clinical population. This behavioural phenotype often manifests in subclinical symptoms, with the absence of a defined diagnosis. According to this conceptualisation, children born preterm are more likely to present socio-emotional and cognitive difficulties, impacting their well-being in the long term.

Socio-emotional competencies refer to a set of skills that allows one to successfully express, understand and deal efficiently with emotions and to successfully interact within a social context. These abilities are crucial for well-being and positive relationships and are predictive of academic success [7,8,9,10,11]. Preterms’ difficulties in this domain include diminished social competencies and social reasoning abilities [12], emotional dysregulation, internalising problems, low self-esteem and behavioural problems [6,13,14]. These problems are already visible in early childhood [15,16,17]. 

Executive functions (EF) are a set of high-order cognitive functions crucial for goal-oriented behaviour and problem-solving. They promote psychological resilience and protect against stressful events that impact mental health [18]. Deficits in these abilities play an important role in explaining emotional and behavioural problems [19] and are largely impacted by premature birth as well [20]. In preterm children, these problems become particularly evident at school age [21,22,23] but are already present in preschoolers [24,25]. Working memory is one of the executive functions frequently impacted in this population, and deficits in this domain have been identified both in the visual [26,27] and verbal modality [28,29], starting from 2 years of age [30].

Emotions and executive functions are tightly interrelated, and both have an important impact on daily functioning and learning [8,9,31]. A growing body of studies has developed intervention programs to train socio-emotional competencies and executive functions in typically and atypically developing children [11,32,33,34,35]. Emotion skills training was found to reduce maladaptive behaviours and improve mental well-being in healthy children and gave promising results in children with developmental disabilities such as autism spectrum disorder [36,37,38].

Regarding executive functions, different training types showed beneficial impacts on inhibition, attention and memory, among others [39,40,41]. In the premature population, different training types for executive functions have been proposed [42,43,44], albeit with unsatisfactory results, especially in the long term. Recently, our group proposed a mindfulness-based training program for very preterm young adolescents, aiming to target both executive functioning and socio-emotional competencies [34]. This randomised controlled trial (RCT) showed a beneficial effect of this intervention on executive, socio-emotional and behavioural competencies, as reported by parent questionnaires and computerised tasks. Long-term benefits of the intervention were visible after three months in only a subgroup of our sample, namely in children with higher gestational age and birth weight [34].

To our knowledge, there are no interventions proposed for younger preterm children targeting both socio-emotional competencies and executive functions. Given the well-known impact of prematurity on these constructs, we decided to implement a computerised intervention that could benefit premature children between 6 and 9 years of age. We targeted children at the beginning of their scholarly path because this period is particularly demanding from both a socio-emotional and executive point of view. It is often at this age that premature children begin to manifest the first emotional and cognitive symptoms that previously remained subclinical.

The intervention selected for this study is the Vis-à-vis program, a 12-week computerised training program developed by Glaser and colleagues [36]. Its goal is to improve socio-emotional functioning and working memory in children with developmental disabilities. Previous research showed that after the training, children with idiopathic developmental delay, autism and 22q11 deletion syndrome showed marked improvements in nonverbal reasoning and facial emotion recognition and less behavioural problems overall [36]. Furthermore, for children with autism and 22q11 deletion syndrome, fMRI assessments conducted before and after training demonstrated that the subsequent improvements were associated with increased BOLD responses in brain regions specialised for face processing [45]. So far, there is no evidence about the efficacy of this training in children born preterm. The reduced executive functioning and emotional competencies associated with prematurity make preterm children the perfect candidates for this type of intervention.

Therefore, the first aim of the current pilot study was to evaluate the feasibility of the Vis-à-vis training in a cohort of very preterm (VPT) children aged between 6 and 9 years old using an RCT design. Our second aim was to evaluate the efficacy of the Vis-à-vis program on socio-emotional and executive functions. We hypothesised that the Vis-à-vis training would be appealing and doable for most of the participants enrolled in the study. Moreover, we anticipated that Vis-à-vis would improve emotion recognition and understanding, theory of mind and executive functioning in our population. This would mean that the Vis-à-vis program holds the potential to be an efficient training approach for improving socio-emotional adjustment and executive functioning in VPT children at the beginning of school, reducing the risk of developing more severe behavioural and cognitive difficulties later.

## 2. Materials and Methods

### 2.1. Participants

**Figure 1:**
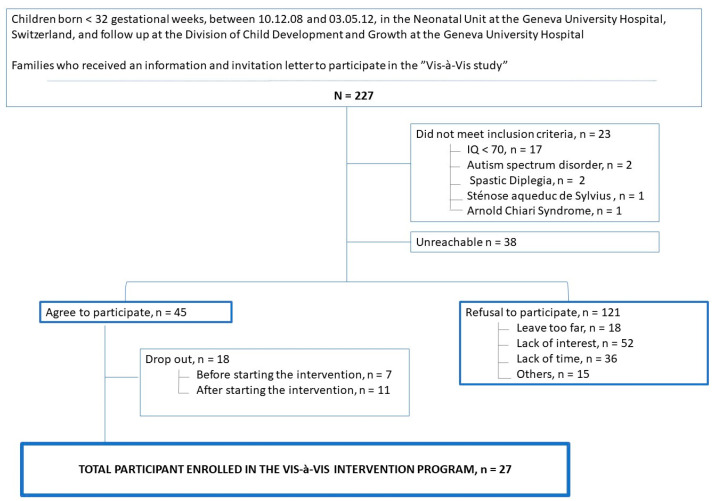
CONSORT diagram detailing participant flow.

A total of 227 very preterm (VPT) children, aged 6 to 9 years, received an informational letter and were invited to take part in the study. These children were born before 32 gestational weeks, between 10 December 2008 and 3 May 2012, at the Neonatal Unit of Geneva University Hospital in Switzerland. Follow-up was conducted at the Division of Child Development and Growth at the same hospital. Exclusion criteria included an intelligence quotient below 70, the presence of severe neurological or sensory disabilities (such as cerebral palsy, blindness or deafness) and insufficient comprehension of French. Figure 1 presents a CONSORT diagram that outlines the participant flow.

This study employed a wedge randomised controlled trial design. It was registered and approved as a Clinical Trial by the Swiss Ethics Committees on research involving humans (ID: 2015-00175) and was conducted in accordance with the Declaration of Helsinki. Data collection took place at Campus Biotech, Geneva, Switzerland. Caregivers provided informed written consent, and participants received a gift voucher worth 100 Swiss francs upon completing the entire protocol.

### 2.2. Procedure

**Figure 2:**
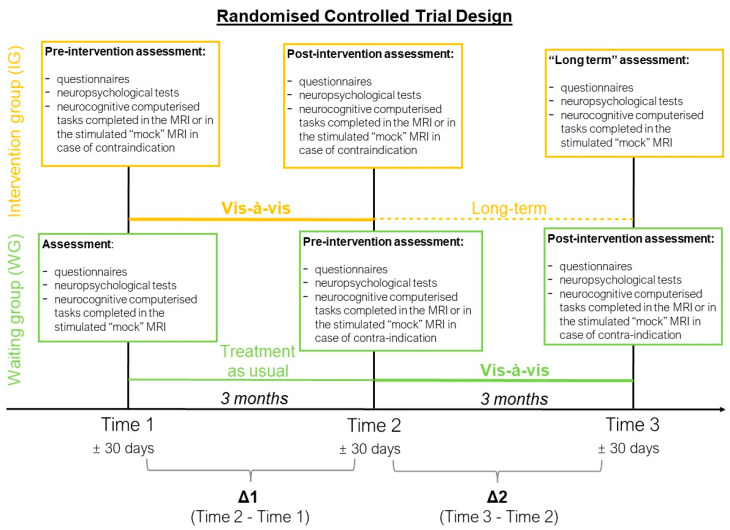
Randomised controlled trial design of the study.

This study used a cross-over RCT design (Figure 2).

After enrollment, families were randomly assigned to either the intervention group (IG) or the waiting group (WG). To achieve this, an independent biostatistician created a random number table. Families were given the next available sequential study number, which matched a sealed envelope containing their randomised assignment to either the IG or WG. After the family consented to participate and before the first appointment, experimenters opened the envelope to reveal the group allocation. Randomisation was performed at the family level instead of for individual participants, ensuring that families with twins could have their children undergo the intervention simultaneously.

As a first step, participants completed a baseline assessment (Time 0) to assess general intellectual functioning and to collect demographic information. The following assessments were completed at three time points (Time 1, Time 2 and Time 3) and comprised neuropsychological test, parent-reported questionnaires and computerised cognitive tasks. Participants allocated in the IG completed the intervention between Time 1 and Time 2, while participants in the WG completed the intervention between Time 2 and 3. All children completed the pre-intervention assessment (Time 1 for the IG and Time 2 for the WG) within one month prior to starting the intervention. The post-intervention assessment (Time 2 for the IG and Time 3 for the WG) was conducted within one month following the end of the intervention. Children in the IG were asked to complete a long-term assessment, represented by the final assessment (Time 3, “long-term assessment”), conducted three months after the post-intervention assessment (Time 2). For the WG group, the remaining assessment (Time 1) was conducted three months before the pre-intervention assessment (Time 2). Assessments were carried out by psychologists and psychology trainees who were aware of the participants’ group allocations.

### 2.3. Neonatal and Demographic Characteristics

Neonatal characteristics such as birth weight, gestational age, presence of multiple births, presence of periventricular leukomalacia (PVL), intraventricular haemorrhage (IVH, grades III and IV) and bronchopulmonary dysplasia (BPD) were retrieved from medical records. PVL, IVH and BPD were defined as previously published [46]. Demographic characteristics were documented through parent-reported questionnaires. The socio-economic status (SES) of families was estimated using the Largo scale [47], a validated 12-point questionnaire assessing maternal education and paternal occupation. Higher scores reflect lower SES. General intellectual functioning was assessed using the Kaufman Assessment Battery for Children, 2nd edition (KABC) [48]. This battery allows us to measure the fluid-crystallized index (FCI), which is composed of five scales assessing visual processing, long-term storage and retrieval, short-term memory, crystallized knowledge and fluid reasoning. This index is considered a reliable measure of the general intelligence factor [49] and has a mean of 100 and a standard deviation of 15.

### 2.4. The Vis-à-Vis Training

The Vis-à-vis (“Face-to-face”) training (VAV) is a software package developed by the Research Unit of the Office Médico-Pédagogique of the University of Geneva [36] for children aged 7 to 16 years. The aim of this computerised training is to improve socio-emotional functioning and working memory in school-aged children with autism and other developmental disabilities. It consists of four 20 min computerised sessions per week for 12 weeks. Children complete the intervention individually but under the supervision of an adult, allowing them to discuss the content of the training with parents or caregivers. The program is available in French and in English. Twice per week, a short teaching module precedes the session and gives a theoretical background about the emotions that will be trained during the session, explaining associated anatomical qualities and giving practical examples using animated cartoon sequences or vignettes. In the exercises involving emotions, the six universal facial expressions and associated mental states are trained (happiness, sadness, anger, fear, disgust, surprise, according to Ekman and Friesen, 1971 [50]). Emotions are associated with specific colour labels (e.g., red for anger, yellow for happiness, etc.), which remain consistent throughout the training to help children who cannot read fluently.

Concerning the structure and content of the intervention, VAV is composed of three modules: focus on the eyes, emotion recognition and understanding, and working memory. Each module contains different exercises in the form of interactive games, each of which is practised twice per week during the training period. Exercises become progressively more difficult throughout the 12 weeks. For all exercises, children answer using the mouse.

The focus on the eyes module aims to train children to focus on the eyes when decoding an emotional expression in order to better recognise the emotion. In the exercises of this module, participants are asked to recognise facial expressions using only the eye region of the face, to match a pair of eyes to the corresponding face or emotions, and to select the appropriate emotion label for an emotional face from a list of options.

The emotion recognition and understanding module trains children’s theory of mind and ability to recognise emotions. In the first exercise, participants are presented with different scenarios of a character, and they are asked to deduct their feeling states according to the different scenarios. This helps them understand that a person’s feelings change according to different situations. In another exercise, children must attribute emotions and facial expressions to different characters in each context and thus learn that people can have different emotional responses to the same event.

Finally, the working memory module includes three visuospatial working memory exercises. The first two tasks are based on the spatial span principle. In the first task, a series of lights turn on and off one by one in a visuospatial grid, and children are asked to reproduce the sequence in order. In the second task, a group of animals is displayed in a randomised order and disappears one at a time. Children must reproduce the disappearing sequence by clicking the mouse in the correct order of disappearance. The last task is a memory exercise involving a set of cards displayed facedown, where children must search for identical pairs.

For all the exercises, children receive positive reinforcement through feedback, and newly learned material is frequently repeated.

### 2.5. Outcome Measures

Measures assessing behaviour and socio-emotional competencies:

Strengths and Difficulties Questionnaire, French Version (SDQ—Parent Version) [51]: The SDQ is a well-validated 25-item questionnaire designed to assess behavioural problems in children and adolescents aged 4 to 16. It consists of five subscales that evaluate emotional symptoms, conduct problems, hyperactivity/inattention, peer problems and prosocial behaviour. Parents respond to each item using a 3-point scale (0 = not true, 1 = somewhat true, 2 = certainly true). The score for each subscale is the sum of the related items. Higher scores indicate more severe difficulties in each domain, except for the prosocial behaviour scale, where a higher score signifies positive behaviour. Raw scores for each subscale were used in the analyses.

Theory of mind (NEPSY II) [52]: The Theory of Mind subtest assesses the capacity to understand mental contents such as beliefs, intentions or deceptions and other’s perspectives. In the first task, different verbal scenarios are verbally presented to the child, and they must answer questions about intentions, beliefs, other’s thoughts, ideas and understanding of figurative language. In the second task, different drawn scenarios are presented to the participants, and they must choose the photograph with the facial emotional expression that appropriately matches the scenario. Raw scores were used in the analyses and referred to as theory of mind.

Affect recognition (NEPSY II) [52]: The Affect Recognition subtest measures the capacity to recognise facial emotional expressions (happiness, sadness, anger, disgust, fear and neutral). It is composed of different tasks, using photographs of children’s faces. In the first one, one photograph is presented at the top of the page, and the participant is asked to find another photograph, among four, that displays the same emotion. In the second task, four photographs are presented, and the participant must choose the two that display the same emotional expression. In the last task, the participant must look at one photograph for 5 s and choose two images that display the same emotion as the one seen before. Raw scores were used in the analyses and referred to as affect recognition.

Measures assessing executive competencies:

Behaviour rating inventory of executive function (BRIEF—Parent Version) [53]: The BRIEF questionnaire assesses attention capacities and executive abilities in daily life. It includes 86 items organised into eight clinical scales: inhibit, shift, emotional control, initiate, working memory, plan/organise, organisation of materials. Parents respond on a three-point scale (never, sometimes, often). Higher scores indicate greater difficulties in executive functioning. Raw scores for each subscale were used in the analyses.

Letter–number sequencing subtest from the Wechsler Intelligence Scale for Children-5th Edition (WISC-V) [54]: This subtest evaluates short-term memory skills in processing and re-sequencing information. The task involves listening to a series of letters and digits and then repeating the stimuli with the letters in alphabetical order and the digits in ascending numerical order. The difficulty increases from two alphanumeric characters per string to eight characters. Each string consists of three items, totalling 21 items. Fully correct responses earn 1 point each, with a maximum possible score of 21 points. Raw scores were used in the analyses.

Flanker visual filtering task [55,56]: This computerised task was used to assess inhibition capacities and the ability to resist interference from visual distractors. Each trial presented a horizontal line of five fish. Participants were instructed to quickly indicate whether the central fish was facing left or right. If all five fish were pointing in the same direction, the trial was congruent; if the four distracting fish were pointing in the opposite direction of the central target fish, the trial was incongruent. An inhibition score was calculated by subtracting the accuracy score in the congruent conditions from the accuracy score in the incongruent conditions. This raw score was used in the analyses.

Orbitofrontal reality filtering task (ORFi)—children version [57,58]: This computerised task assesses recognition memory and reality filtering, which is the ability to decide whether a memory is pertinent to the ongoing reality or not. It is a continuous recognition task composed of two runs in which the same set of drawings is presented on the screen and repeated once. For each image, participants are asked to indicate if they have already seen the image within the ongoing run or not. The first run assesses recognition capacity, while the second one measures reality filtering. The temporal context confusion index (TCC), defined as the relative increase of false positives in the second over the first run [59], was used as a measure of reality filtering and was included in the analyses.

### 2.6. Statistical Analyses

All analyses were conducted using R software, version 3.5.2 (Team, R. Boston, MA, USA, 2020), following the intention-to-treat principle [60]. Consequently, data from all 45 VPT children enrolled in the study were included in the analyses. For each measure and each participant, raw scores were used to calculate the differences between Time 1 and Time 2 (Time 1 − Time 2 = Δ1) and between Time 2 and Time 3 (Time 3 − Time 2 = Δ2), see Figure 2. A positive Δ indicates an increase in the score between the two time points, whereas a negative Δ indicates a decrease in the score between the two time points. The effect of the Vis-à-vis intervention was evaluated using linear regression models. Assumptions of linear regression models were assessed based on visual inspections of the residuals’ distribution. The interaction of time (i.e., Δ1 and Δ2) by group (i.e., IG and WG) was modelled as independent variables, and fixed effects of outcome measures were modelled as dependent variables. If the *p*-value of the model was significant, planned contrasts were used to compare outcome measures between the different levels of the independent variables time and group. The effect of the intervention immediately after the Vis-à-vis training was assessed using the planned contrast defined as “Vis-à-vis” (i.e., Δ1 of IG and Δ2 of WG) versus “treatment as usual” (i.e., Δ1 of WG). The delayed effect of the intervention was assessed using the planned contrast defined as “long-term” (i.e., Δ2 of IG) versus “treatment as usual” (i.e., Δ1 of WG). When the effect of the intervention right after the Vis-à-vis training was significant (“Vis-à-vis” vs. “treatment as usual”), the long-term effect of the intervention was assessed using the planned contrast defined as “Vis-à-vis” (i.e., Δ1 of IG and Δ2 of WG) versus “long term” (i.e., Δ2 of IG). The *p*-values were corrected for multiple comparisons using the Benjamini and Hochberg method, which controls the False Discovery Rate (FDR, q values ≤ 0.05) [61].

## 3. Results

### 3.1. Neonatal and Demographic Characteristics of the Sample

Two hundred and twenty-seven eligible VPT children were invited to participate in the study between May 2017 and June 2019. The final acceptance rate for participation was 20%, resulting in a total of 45 children enrolled, randomly divided between the IG (n = 25) and the WG (n = 20). Of note, due to difficulties in recruitment and our strict inclusion criteria, we were unable to enrol 60 participants as initially planned for the randomisation process. As a result, the final randomisation did not yield an equal number of participants per group, resulting in 25 children in the IG and 20 in the WG.

Following enrolment in the study, there was a dropout rate of 40%, resulting in 18 participants discontinuing their involvement before the start of the training due to lack of motivation or time. Finally, 27 VPT children, corresponding to 12% of the initially eligible participants, successfully completed both the Vis-à-vis training and all follow-up assessments (Figure 1).

Neonatal and demographic characteristics of the 45 participants enrolled in the RCT study (IG, n = 25; WG, n = 20) are shown in Table 1 and Table 2.

The mean birth weight was 1320 g (SD = 345.29), and the mean gestational age was 29.83 weeks (SD = 1.67). Regarding the neonatal characteristics, there were no significant differences between the IG and the WG for birth weight, gestational age, presence of cystic periventricular leukomalacia, intraventricular haemorrhage (grade III and IV) and bronchopulmonary dysplasia. Concerning the demographic characteristics, parents’ nationality and native language were in accordance with the general population of the Geneva area, with most parents being Swiss or French and speaking French as their native language. No significant difference was found for sex, age at testing, FCI index, socio-economic status of the parents as well as parents’ nationality and native language. Parent-reported questionnaires were administered to gather additional information on the child’s socio-demographic background, overall health, interventions, schooling and friendships (see Supplementary Table S1). Notably, 64% of children in the IG group (n = 16 out of 25) and 60% of children in the WG group (n = 12 out of 20) had received therapy or daily intervention, whether past or ongoing. There was no significant difference between the groups in the proportion of children receiving past or ongoing therapy or daily intervention (X^2^ (1, N = 45) = 0.129, *p* = 0.719).

### 3.2. Compliance

Among the 227 eligible VPT children, 45 (20%) were enrolled in the study. Among this group, 7 (15%) withdrew before the intervention began, and 11 (24%) dropped out after the intervention commenced. As a result, 27 (60%) VPT children completed the entire training along with assessments at all four designated time points. As our study adheres to the intention-to-treat principle, we included all 45 initially enrolled children in the analysis.

### 3.3. Main Outcomes

#### 3.3.1. Behavioural and Socio-Emotional Outcomes

Linear regression analysis adjusted for multiple comparisons showed a significant effect on the affect recognition score of the NEPSY (F (3,56) = 5.45, q = 0.036). The planned contrast “VAV” versus “treatment as usual” showed an increased affect recognition score immediately after the intervention (q = 0.018), suggesting that the VAV training led to an enhanced capacity for recognising emotions. The planned contrast “VAV” versus “long term” showed a decreased affect recognition score three months after the intervention (q = 0.007), denoting that improvements obtained immediately after training are not maintained over the long term. The delayed effect of the intervention (planned contrast “long term” versus “treatment as usual”) was not significant (q = 0.866). There were no other significant effects of the training on other behavioural and socio-emotional scores, represented by the five sub-scores of the SDQ parent-reported questionnaire and the theory of mind score of the NEPSY-II (Table 3).

#### 3.3.2. Executive Competencies Outcomes

There was no robust effect of the VAV intervention on scores evaluating executive competencies, including scores based on parent-reported questionnaires (i.e., the eight sub-scores of the BRIEF questionnaire) and on neuropsychological testing (i.e., the working memory score assessed using the letter–number sequencing task from the WISC-IV, the flanker inhibition score and the reality filtering assessed with the TCC, Table 3).

## 4. Discussion

In this pilot study, we aimed to estimate the feasibility of the Vis-à-vis program—a computerised training targeting socio-emotional competencies and working memory—in VPT school-aged children, using an RCT design. We postulated that this kind of training would be appealing and doable for most of the participants enrolled in the study. In addition, we hypothesised that Vis-à-vis would improve emotion recognition and understanding, theory of mind and executive functioning in our population. To the best of our knowledge, this is the first computerised training proposed to VPT children in the first years of school to mitigate the impact of prematurity on both socio-emotional and executive skills.

In terms of feasibility, the acceptance rate (20%) was satisfactory, considering the length of the training and the weekly commitment required (four sessions per week for 12 weeks). However, the dropout rate was relatively high (40%) and warrants some considerations.

Among the 18 participants who dropped out, 7 were in the waiting group and decided to withdraw from the study before commencing the training. While we did not document the reasons for these dropouts, it is plausible that the extended waiting period (approximately 12 weeks) before starting the training negatively impacted their motivation and adherence to the study protocol. This underscores the importance of providing clear and thorough information about the study timeline, potential challenges, as well as support mechanisms during recruitment to enhance participant retention. Moreover, this also suggests that future studies should consider shorter waiting periods or engaging activities during the waiting phase to maintain participant interest. Ideally, the inclusion of a control or placebo condition could help participants and their families feel continuously engaged and supported throughout the study. In addition, among the 18 dropouts, 11 left the study after the training had begun. The main reasons cited were a lack of time, technical issues related to the online program or the perception that the games were too easy and, hence, boring for children. In our opinion, the computerised, remotely administered format of the Vis-à-vis program has both strengths and possible weaknesses. The online format of the training did not require any software to be installed on participant’s computers, facilitating the set up. In addition, this format provided families with substantial independence and flexibility in choosing when to complete the training sessions, aligning with their schedule. However, this mode of training also required that participants and caregivers process basic computer proficiency, a criterion met by our sample. Nevertheless, it is worth noting that we encountered several technical problems linked to the accessibility of the website, and this surely interfered with adherence to the program. In addition, even if a remote intervention provides ease of administration, the absence of face-to-face interactions may have made it more difficult for researchers to keep in touch with participants. However, a strong point of the Vis-à-vis program is that it allows providers to monitor progress online, giving detailed information on sessions’ completion. Based on this data, it can be inferred that children who completed the training until the end had very high compliance, as they finished all the training sessions and the required assessments.

With respect to the training outcomes, we found a significant improvement in emotion knowledge and recognition immediately after the intervention, reflected by higher performances in the affect recognition subtest of the NEPSY. This finding is in line with previous studies suggesting that emotion comprehension can be trained in young children [10]. However, this result was not maintained in the long term, i.e., 3 months after the end of the intervention, and no effect was found on measures of theory of mind. Again, we believe that the characteristics of the training program might account for why the observed benefits do not last over time and fail to encompass other social competencies. In a previous study conducted by Ornaghi and colleagues [62], they designed a conversational intervention focusing on emotions with the goal of enhancing emotional understanding, theory of mind and empathy. Their intervention was found to be effective, with the positive effects persisting for more than 6 months. We speculate that the absence of direct face-to-face interaction and engagement between participants and researchers could explain why our results do not exhibit the same long-term sustainability.

Furthermore, no significant effect was found on parent-reported questionnaires assessing executive functions and behavioural problems. This finding is unsurprising, as detecting these kinds of alterations within a relatively brief timeframe (12 weeks) can be challenging. Parents typically only report differences in children’s behaviour and executive functions across time when substantial changes are observed, which was not the case after the VAV intervention. Additionally, no effects were found on computerised tasks evaluating memory and EF (reality filtering task and flanker task). The existing literature provides mixed evidence regarding the benefits of computerised EF training in preterm children [63], with most studies having small sample sizes and lacking randomisation. It is also plausible that improvements in certain skills related to the training might have become evident after more than 3 weeks of time. For instance, Lee et al. [64] found an effect of cognitive training on verbal WM in preterm children only 5 weeks after the end of the training. Similarly, Holmes and colleagues [65] found a training-related effect on mathematical reasoning only after 6 months. Therefore, conducting a more extended long-term follow-up might have revealed effects on the outcome measures of interest. In addition, it is important to acknowledge that there can be interindividual variability in response to this kind of intervention [66,67]. Our previous study [34] showed different effects of a mindfulness-based intervention in two distinct subgroups of participants based on prematurity level. This highlighted the possibility that certain individuals may derive greater benefits from the training than others at different time points. Unfortunately, our small sample did not allow this kind of analysis.

In summary, implementing this type of computerised training in VPT children of this age range is feasible; however, given the high dropout rate, overall compliance remains unsatisfactory. Overall, compliance results corroborate former research using computerised training in preterm children. Previous studies in this population mainly aimed to improve executive function and showed low levels of recruitment rate and training compliance [44,63,64]. The computerised nature of the training and the absence of interaction with the researchers could explain this result. Indeed, an in-person training set up by our group with VPT young adolescents yielded more encouraging results, with an acceptance rate of 38% and a drop-out rate of 17% before the start of the training and none of the young adolescents who dropped out after the training commenced [34].

Finally, our study employed a gold standard RCT design, and the data were analysed using an intention-to-treat approach. However, several theoretical and methodological limitations should be acknowledged to inform future research. First, the absence of an active control or placebo condition is a significant limitation. When control groups do not engage in any new or stimulating activities, such as in a waitlist control comparison, improvements observed in the intervention group may be attributed to nonspecific effects of novelty rather than the intervention itself [68]. Therefore, comparing the Vis-à-Vis intervention with an equally engaging active control condition is necessary to provide reliable results and to better understand the factors contributing to the intervention’s beneficial effects. Implementing an active control group could also mitigate the high dropout rate observed in the WG of the current study. Secondly, an active control condition would enable participants and their families to remain unaware of the treatment allocation, thereby increasing the validity of the results. This approach would also help isolate the specific effects attributable to the Vis-à-Vis intervention. Additionally, factors such as the home environment, caregiver involvement and motivation to engage in the training were not considered in our study [69,70]. These factors could impact the intervention outcomes and should be accounted for in future research.

## 5. Conclusions

This pilot study is the first to evaluate the efficacy of a computerised training on emotional and executive competencies in VPT school-aged children. Our results suggest a positive effect on emotion capacities immediately after the Vis-à-vis training but no effects on other domains. Our sample size being limited due to the low attrition rate, these findings warrant further exploration with a larger sample before we can recommend Vis-à-vis as effective training for VPT early school-age children. Consequently, future studies should investigate individual differences in training outcomes, to confirm the usefulness of this training to palliate the negative consequences of prematurity and ameliorate the outcome of this population.

## Figures and Tables

**Table 1:** Neonatal characteristics of VPT children enrolled in the RCT (n = 45) and statistical comparison between the intervention group (IG) and the waiting group (WG).

	RCT, n = 45		
	Intervention Group (IG) n = 25	Waiting Group (WG) n = 20	Group Comparison (IG vs. WG)
Birth weight, mean (SD) in grams	1306.80 (331.06)	1335.00 (358.98)	t(43) = −0.462, *p* = 0.646, q = 0. 821
Gestational age, mean (SD) in weeks	29.72 (1.46)	29.95 (1.88)	t(43) = −0.274, *p* = 0.786, q = 0.821
Multiple births, n (%)	5 (20%)	11 (57.9%)	Χ^2^(1) = 6.699, *p* = 0.01, q = 0.06
cPVL, n (%)	0	0	χ^2^(1) = 0.903, *p* = 0.342, q = 0.684
IVH Grades III and IV, n (%)	1 (4%)	1 (5%)	χ^2^(1) = 2, *p* = 0.157, q = 0.471
BPD, n (%)	7 (28%)	5 (25%)	χ^2^(1) = 0.051, *p* = 0.821, q = 0.821

Abbreviations: cystic periventricular leukomalacia = cPVL, intraventricular haemorrhage = IVH, bronchopulmonary dysplasia = BPD; independent-sample *t*-test and Chi-square tests, as appropriate, were employed to compare the randomised groups

**Table 2:** Baseline demographic characteristics of VPT children enrolled in the RCT (n = 45) and statistical comparisons between intervention group (IG) and waiting group (WG).

		RCT, n = 45		
		Intervention Group (IG) n = 25	Waiting Group (WG) n = 20	Group Comparison (IG vs. WG)
Sex	Female, n	13 (52%)	12 60%)	χ^2^(1) = 0.288, *p* = 0.592, q = 0.915
	Male, n	12 (48 %)	8 (40%)	
Age at baseline, mean (SD) in months		95.48 (14.68)	95.94 (14.49)	t(39) = 0.098, *p* = 0.922, q = 0.922
FCI, mean (SD)		105.92 (13.33)	104.07 (14.96)	t(38) = 0.407, *p* = 0.686, q = 0.915
Socio-economic status (SES), mean (SD)		4.64 (2.06)	3.61 (2.17)	t(41) = 1.58, *p* = 0.122, q = 0.912
Mother’s nationality	Swiss, n (%)	13 (52%)	7 (35%)	χ^2^(2) = 1.592, *p* = 0.451, q = 0.912
	French, n (%)	7 (28%)	3 (15%)	
	Other, n (%) Unknown, n (%)	5 (20%) 0 (0%)	6 (30%) 4 (20%)	
Father’s nationality	Swiss, n (%)	13 (52%)	9 (45%)	χ^2^(2) = 1.571, *p* = 0.456, q = 0.912
	French, n (%)	5 (20%)	1 (5%)	
	Other, n (%)	7 (28%)	6 (30%)	
	Unknown, n (%)	0 (0%)	4 (20%)	
Mother’s native language	French, n (%)	16 (64%)	11 (55%)	χ^2^(1) = 0.019, *p* = 0.89, q = 0.922
	Other, n (%)	8 (32%)	5 (25%)	
	Unknown, n (%)	1 (4%)	4 (20%)	
Father’s native language	French, n (%)	17 (68%)	8 (40%)	χ^2^(1) = 0.86, *p* = 0.354, q = 0.912
	Other, n (%)	8 (32%)	7 (35%)	
	Unknown, n (%)	0 (0%)	5 (25%)	

Note: The Fluid-Crystallized Index (FCI) from the Kaufman Assessment Battery for Children—2nd Edition (K-ABC-II) was employed to assess general intellectual functioning. Independent-sample t-tests or Chi-Square tests were used to compare the intervention group and the waiting group.

**Table 3:** Results of the linear model on the effect of the intervention (IG, n = 25; WG, n = 20).

	df	F	*p*-Value	q < 0.05
WISC—IV				
Working memory (LNS)	F (3,56)	1.393	0.254	0.976
**NEPSY—II**				
Theory of mind	F (3,56)	0.938	0.429	0.976
Affect recognition	F (3,56)	5.448	**0.002**	**0.036**
BRIEF—Parent form				
Inhibition	F (3,54)	0.096	0.962	0.999
Shift	F (3,54)	0.744	0.531	0.9756
Emotion control	F (3,54)	0.031	0.999	0.999
Initiate	F (3,53)	0.598	0.619	0.999
Monitoring	F (3,54)	0.728	0.539	0.976
Organisation	F (3,52)	1.569	0.208	0.976
Planification	F (3,53)	0.338	0.798	0.999
Working memory	F (3,54)	3.588	0.019	0.171
SDQ				
Hyperactivity/Attention	F (3,56)	1.111	0.353	0.976
Emotional difficulties	F (3,56)	0.272	0.846	0.999
Conduct problems	F (3,56)	0.085	0.968	0.999
Peer problems	F (3,56)	0.517	0.672	0.999
Prosocial behaviour	F (3,56)	1.198	0.318	0.976
Flanker				
Inhibition	F (3,35)	0.728	0.542	0.976
Reality Filtering				
TCC score	F (3,49)	0.221	0.882	0.999

Abbreviation: LNS: letter–number sequencing. All *p*-values from linear models that survived the false discovery rate (FDR) correction as per Benjamini and Hochberg (1995) are shown in bold (q < 0.05).

## Data Availability

Data are available on request due to restrictions. The data presented in this study are available on request from the corresponding author. The data are not publicly available due to ethical restrictions.

## Supplementary Materials

The following supporting information can be downloaded at: https://www.mdpi.com/article/10.3390/children11080956/s1, Table S1: Parents’ questionnaire on their children schooling, friendship, medical history and global health. Table S2: Self-reported questionnaire on schooling, friendship, activities and family organisation.
